# Supplementary material for: Long-Term Effects of Rifaximin on Patients with Hepatic Encephalopathy: Its Possible Effects on the Improvement in the Blood Ammonia Concentration Levels, Hepatic Spare Ability and Refractory Ascites
Source: Medicina (Kaunas). 2022 Sep 14;58(9):1276. doi: 10.3390/medicina58091276 (PMC9501622; doi:10.3390/medicina58091276)
Supplement: Supplementary file 1 [file medicina-58-01276-s001.zip › medicina-1864087-supplementary.pdf]

Supplementary Table S1. Clinical characteristics of the 27 improved hepatic ascites cases.

No	Age	Sex	Etiology	Added drug or therapy for hepatic ascites after the administration of RFX
1	69	M	NASH	Tolvaptan, Propranolol
2	81	F	NASH	Tolvaptan, BCAA, Carnitine
3	74	F	HCV	Tolvaptan, BCAA
4	67	M	HBV	Tolvaptan, BCAA
5	74	M	NASH	Tolvaptan
6	43	M	Alcoholic	Tolvaptan, BCAA, Carnitine, Zinc
7	61	F	AIH+PBC	Tolvaptan, Spironolactone, BCAA, Carnitine, Synthetic disaccharides
8	66	M	Alcoholic	Tolvaptan, Furosemide, BCAA, Carnitine
9	55	M	Alcoholic	Tolvaptan, Synthetic disaccharides, Carnitine
10	50	M	Alcoholic	Furosemide, BCAA
11	61	F	Alcoholic	Furosemide, Spironolactone, BCAA, Carnitine, Zinc, Abstinence
12	84	M	Cryptogenic	Spironolactone, Zinc, Synthetic disaccharides
13	67	M	Alcoholic	Spironolactone, Zinc, Abstinence
14	52	M	Alcoholic	Steroid, BCAA
15	61	M	HCV	Synthetic disaccharides, PSE performing
16	63	M	HCV+ Alcoholic	BCAA, Carnitine, Abstinence
17	27	F	Alcoholic	Zinc, Abstinence
18	69	M	HCV+ Alcoholic	Abstinence
19	69	M	Alcoholic	Abstinence
20	65	M	Alcoholic	Abstinence
21	62	M	Alcoholic	Abstinence
22	70	M	Alcoholic (abstinence >5 years)	None
23	74	M	HCV	None
24	62	M	HCV	None
25	62	F	NASH	None
26	74	F	Cryptogenic	None
27	76	F	NASH	None

AIH, autoimmune hepatitis; BCAA, branched chain amino acid; F, female; HBV, hepatitis B virus; HCV, hepatitis C virus; M, male; NASH, nonalcoholic steatohepatitis; PBC, primary biliary cholangitis; PSE, partial splenic embolization; RFX, rifaximin

Supplementary Table S2. Relationship between hepatic ascites control 3 months after RFX administration and Child–Pugh class (*n* = 97).

	**Satisfactory control**	**Poor control**
Child–Pugh class A and B	36	24
Child–Pugh class C	12	25

***n*,** number
